# Will an App Fill the Gap? Innovative Technology to Provide Point-of-Care Information

**DOI:** 10.3389/fpubh.2014.00009

**Published:** 2014-02-05

**Authors:** Patricia Codyre

**Affiliations:** ^1^Institute of Public Health, University of Heidelberg, Heidelberg, Germany

**Keywords:** mobile application, information communication technology, emerging technology, clinical decision support systems, medical informatics, patient adherence

The use of mobile devices has become increasingly universal in almost every aspect of life. Whether it is for communication, web browsing, or recreation, they are progressively being seen as complimentary – if not replacing – personal computer as the static predecessor [see the world-wide mobile subscriptions from 2013 ICT facts and figures in Ref. (1); Figure A1 in Appendix]. But mobile devices are not limited to the personal space. We have now seen their emerging use in healthcare, which encompasses diverse issues to solve clinical problems and promote public health initiatives in pioneering ways. The role of information science in medicine continues to expand as it merges into the mainstream of clinical practice. Since 2009, the World Health Organization (WHO) has coined the term mHealth as “medical and public health practice supported by mobile devices, such as mobile phones, patient monitoring devices, personal digital assistants (PDAs), and other wireless devices” (2). The essence of mHealth is the exchange of information remotely, whether it is audio, image, medical record, or commands. Expanding access to the latest medical research at the point-of-care to communicating with physicians globally in real time, medicine is now practiced in a technological era. Pew Research Center indicated that 56% of medical practitioners already use mobile applications in clinical setting (3) while another study estimated that 81% of physicians use smartphone by 2012 (4). This is probably because smartphones and tablets are ideal for the mobile nature of medicine due to their portability, ability to enable targeted treatment, and support patient adherence to medication.

Many medical institutes have already incorporated this technology in their curriculum (3), and preliminary data indicate that “doctors who were trained to use a smartphone app for teaching advanced life support had significantly improved scores during … simulation testing” (5). This also underscores the potential use of mobile devices applied to different educational models and learning theories; i.e., from instruction to investigational education, inquiry learning, action research, and communities of practice (CoP). This change in educational content will also result in a *de facto* change in medical practice resulting in an increased focus on communication, quality improvement tools, and acquisition of clinical data.

Medical applications (apps) are also viewed as emerging tools in evidence-based medical practice at the point-of-care (4). The intersection of this technology with medicine is likely to result in efficient access to medical information/data, avoiding unnecessary testing, and saving of both cost and time to busy hospitals and doctors. It is now apparent that mobile devices are an integral part of medical education, patient care, and communication (5). But ultimately, the real benefit of medical mobile technology lies in higher quality of healthcare for the patient.

Mobile apps have approached the intersection of technology and public health and provide promising practices and lessons learned, as well as novel and innovative approaches of how these devices can support medical professionals. Clinical intervention and associated technology must always aim to improve or promote health or health service and quality. The uses of medical apps by the healthcare workforce broadly encompass the following areas (4, 6–8):

- medical apps designed to “improve diagnosis, investigation, treatment, monitoring, and management of disease” (9)
- apps to deliver treatment and interventions designed to improve treatment compliance
- apps for disease management programs and health promotion
- apps to improve health care logistics such as appointment attendance, result notification, or vaccination reminders.

To highlight the mobile nature of both healthcare and smartphone, we showcase the use of mobile devices and applications in surgery and surgical care. Table 1 provides a brief survey of medical apps that were reviewed in the literature and indicates that the presence of mobile technology in surgery now extends beyond medical information retrieving to places such as pre-operative consultation, operating theater, and post-operative care (10, 11). Warnock advises that “as mobile communications and related apps proliferate, it is essential for surgeons to remain well-informed” about how to use this technology to facilitate daily work and what kind of new point-of-care knowledge can be obtained (12). Generally, medical apps are available – from free of change to a special subscription fee – on all popular platforms such as Apple iOS, Android, and Blackberry or Windows (13). Based on apps available between 2009 and 2010, Mosa and coworkers report that the “iOS is the most popular platform for healthcare smartphone applications” but this situation may change as the popularity of the other ones rise (4). A limitation of almost all studies lies in the fact that they examined only apps available in English. Most of the apps are available on the smartphones, but tablet computer is also being increasingly used for “surgical consultations, operating theaters, post-operative care, and surgical education … mainly in thoracic, orthopedic, and ophthalmic surgery where it was shown to improve surgical performance and safety” (3). While Franko claims that medical apps for textbook/reference and techniques/guides are highly requested by orthopedic surgeons (14, 15), rigorously peer-reviewed scientific literature is still found to be most influential for their decision-making process (16).

**Table 1:** **Mobile application in surgery**.

Item	Apps	Feature
1	Epocrates	Interactive “important portable reference and educational tool” (5, 10, 12)
2	AO surgery reference	
3	Zollinger	Medical electronic books (ebooks) for surgeons both for medical reference and education (13, 14)
4	Campbell’s orthopedics	
5	Oxford handbook of clinical surgery	
6	Surgical instrument	A game for learning about medical tools (13)
7	SurgAware	Patient’s pre-operative education (12)
8	3D brain	
9	OsiriX HD	Pre-operative or intraoperative imaging (12)
10	Mobile MIM	
11	SurgiChart	Surgical record log (3)
12	British national formulary	Perioperative and post-operative patient management (13)
13	WHO surgical safety check	A checklist for use in the operating room to improve safety and reduce complication (12)
14	PreOpEval	An “algorithm for pre-operative cardiac assessment … and investigations” (3)
15	Heart surgery risk	An app for “coronary artery-bypass grafting patients that identifies potential … mortality associated with this procedure” (12)

Effective delivery of healthcare services will not be able to depend only, as it does currently, on the clinical decision-making capacity and reliability of self-directed health workers. The medical error rates described in recent national studies demonstrate a collapsing paradigm of the traditional model that an individual practitioner’s accumulated personal experience and judgment is the pinnacle of medical effectiveness (17). The emerging use of apps and mobile technologies in the healthcare setting has the potential to improve patient safety and reduce this alarming trend. Point-of-care medical apps can provide the most current literature and techniques relevant to health care delivery, thus increasing the quality of medical care. However, the development and use of healthcare apps present challenges. One of the primary issues, as a direct result of the proliferation of digital medical knowledge and its effect on medical decision-making, is the retraining of current health care personnel (18). Retraining is more complex than simple computer literacy and encompasses knowledge of the principles of information retrieval, clinical epidemiology, biostatistics, and how to critically appraise the published literature (18). A systematic approach to the retraining of mid-career professionals may assist long-term medical education results. Financial rewards or regulatory requirements that mandate documentation of the sources used to justify and support a particular diagnostic or therapeutic decision could hasten the more widespread use of clinical decision mobile technology. The access and usage of medical apps for point-of-care knowledge will also necessarily mean an inevitable redistribution of power over the decisions that affect the delivery of health care. In health promotion, the concept of power can be defined as the ability to create or resist change, and this is an important foundation for individual and community health workers (19). Patient empowerment will lead to a continuing evolution in the relationship between providers and patients. By enabling people to empower themselves, health promoters can provide the capacity for the individual or community to change their lives and their living conditions, and therefore their health (19).

Interestingly, recent evidence shows that mHealth can enable behavior change and improve health outcomes in resource-limited settings. For example, a “survey among medical residents in Botswana showed how a smartphone pre-installed with medical apps can be an effective way to obtain information in a resource-poor region” (5). While emerging economies face multiple socio-economic, technological, and environmental challenges, there are also multiple opportunities available to assist them in their path to development (9). Some of these opportunities stem from the adaptation of capital, technology, and knowledge to community needs. Mobile communications have emerged as a key technology to bridge the digital divide and as a means to accelerate the dissemination of healthcare information throughout their territories. Emerging studies have shown positive impact of mHealth solutions in resource-poor or low health-literate environments but more evidence, of better quality, is needed to make the health and investment case for future development (20–22). The success of apps and mHealth interventions through Primary Health Care systems is grounded on a high level of community involvement. In areas “where people have limited access to formal health care, increasing coverage for control and prevention of many major diseases require novel approaches” (23). What is essential in the long-term is impartial empirical research, “comparing the cost and efficacy of different systems in well-defined tasks and contexts” (24).

Few would have anticipated how mobile devices have evolved, let alone how it influences healthcare providers and patients. Mobile devices have the potential to fulfill its real promise to facilitate the communication of information for clinical care. The ultimate goal is to provide a set of tools that allows clinicians to access up-to-date guidelines and apply them to the management of patients. Emerging technologies have unlimited potential while we can only surmise on the type of impact apps and mobile devices will have on the future of medicine. If utilized to their maximum potential, apps can support and monitor health improvements at scale and to accelerate achievement of clinical healthcare goals. The converse also holds true; it must be underlined that, if used ineffectively, advancement of these tools could result in medical harm or a waste of resources.

Mobile devices and their applications are perhaps the most dynamic trend in medicine. By 2015, it is predicted that 500 million smartphones around the world will be used for mHealth service (25). From the physician’s point of view, they are increasingly satisfied with medical applications for accessing healthcare information and for clinical practice. Medical professional have found mobile technology to enhance their decision-making process and reduce medical error. The need for new medical applications will continue to rise, as many websites are still incompatible with the mobile web browser (26). As this occurs, the medical community will be inundated by new apps that pop up daily, which can lead to essential medical information to be either so overloaded or fragmented that one simply cannot navigate through (25). Almost all the literature examined point to this serious problem: the contents of current healthcare apps are not always reviewed by medical community and authority, and no high-quality data exists to rigorously evaluate this technology (4, 5, 9, 27). Moreover, other concerns involve doctor–patient personal interaction, data security, and the safety of introducing mobile devices into operating room (3, 4, 12). Another pivotal issue for the deployment of mHealth “lies in establishing best practices which are both cost-effective and supported by rigorous research and evaluation. Policy-makers and funders must promote, legislate, and fund programs that integrate and build upon a common mHealth framework” (28). Additional problems confronting mHealth are the establishment of a platform for knowledge sharing, designing mHealth systems, conducting professional training or integrating mHealth, and building information and communications technology into the health systems of the future. Yet, the challenge of making these services affordable still remains.

To guarantee applicable use of apps and associated devices, implementation must be guided by evidence from evaluation at all design and scale-up stages. The power of this technology will 1 day live up to its promise to help deliver a more advanced, more personalized, and ultimately a better healthcare system.

## Author Contributions

PC conceived the idea, performed the research, wrote, and approved the manuscript.

## Figures and Tables

**Figure A1:**
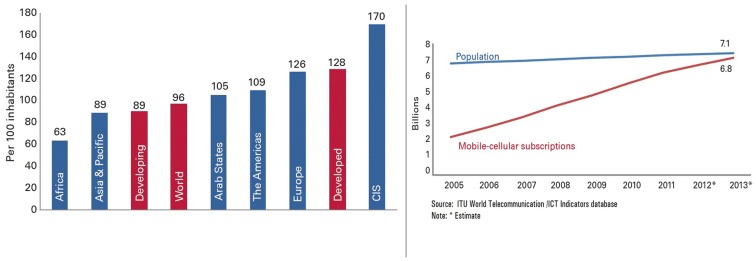
**Active mobile-broadband subscriptions per 100 inhabitants, by region**.
